# Two-Year Mediterranean Diet Intervention Improves Hepatic Health in MASLD Patients

**DOI:** 10.3390/foods14101736

**Published:** 2025-05-14

**Authors:** Margalida Monserrat-Mesquida, Cristina Bouzas, Silvia García, David Mateos, Miguel Casares, Lucía Ugarriza, Cristina Gómez, Antoni Sureda, Josep A. Tur

**Affiliations:** 1 Research Group on Community Nutrition & Oxidative Stress, University of Balearic Islands-IUNICS, 07122 Palma de Mallorca, Spain; margalida.monserrat@uib.es (M.M.-M.); cristina.bouzas@uib.es (C.B.); cristina.gomez@ssib.es (C.G.);; 2 Health Research Institute of the Balearic Islands (IdISBa), 07120 Palma de Mallorca, Spain; 3 CIBER Fisiopatología de la Obesidad y Nutrición (CIBEROBN), Instituto de Salud Carlos III (ISCIII), 28029 Madrid, Spain; 4 Radiodiagnosis Service, Red Asistencial Juaneda, 07011 Palma de Mallorca, Spain; 5 C.S. Camp Redó, IBSalut, 07010 Palma de Mallorca, Spain; 6 Clinical Analysis Service, University Hospital Son Espases, 07120 Palma de Mallorca, Spain

**Keywords:** MASLD, Mediterranean diet, hepatic health, intrahepatic fat content, fatty liver index

## Abstract

***Background:*** Metabolic Dysfunction-Associated Steatotic Liver Disease (MASLD) is one of the leading causes of chronic liver disease, affecting 30% of the global adult population and continuing to rise. ***Objective:*** We aimed to assess the effect of a two-year follow-up Mediterranean diet intervention on parameters of liver health in MASLD patients. ***Methods:*** Sixty-two people between 40 and 60 years of age, all diagnosed with MASLD, were enrolled in the two-year clinical trial, who were randomly assigned to one of three interventions following the Mediterranean diet pattern and the promotion of physical activity. After the intervention, the participants were categorized into two groups according to their progress in adhering to the Mediterranean diet (MedDiet), which was assessed at four follow-up time points, conducted at the start of this study and after 6, 12, and 24 months of intervention. A multivariate general linear model adjusted for age, sex, and intervention (diet and physical activity) was used. Bonferroni’s post hoc test identified differences between groups and sessions within the same group. ***Results:*** Participants in the highly adherent group showed significantly stronger improvement in anthropometric measures, lipid profile, and liver enzyme levels during the follow-up period, along with a reduction in the Dietary Inflammatory Index, intrahepatic fat content, the fatty liver index, and plasma cytokeratin-18 levels compared to baseline. The progress observed in several parameters at 12 months came to a standstill, likely because of the COVID-19 pandemic at that time. At 24 months, following the COVID-19 pandemic, these parameters improved as a result of better adherence to the Mediterranean diet. ***Conclusions:*** Greater adherence to the Mediterranean diet, along with increased physical activity, significantly enhances liver health markers in individuals with MASLD. These findings support the Mediterranean lifestyle as an effective non-pharmacological strategy to improve liver health and prevent liver-related complications in MASLD patients, potentially reducing the future public health burden.

## 1. Introduction

Metabolic Dysfunction-Associated Steatotic Liver Disease (MASLD), recently adopted to replace non-alcoholic fatty liver disease (NAFLD), better reflects the metabolic origins and features of the condition [1]. MASLD is a major cause of chronic liver disease (CLD), impacting approximately 30% of the global adult population, with its prevalence continuing to increase [2]. MASLD encompasses a spectrum of liver disorders characterized by excessive fat buildup in the liver, closely linked to obesity, dyslipidemia, type 2 diabetes, and insulin resistance [3,4].

Adherence to the Mediterranean diet (MedDiet) has shown beneficial effects in managing obesity, cardiovascular disease, and metabolic syndrome (MetS), conditions frequently associated with MASLD. These benefits include reductions in the body mass index, improved lipid profiles, lower blood pressure, and decreased intrahepatic fat content [5,6,7]. Lifestyle interventions in patients with MASLD, particularly a Mediterranean lifestyle based on following a MedDiet and engaging in regular physical activity, has been described as an effective treatment to prevent and reverse MASLD, reducing intrahepatic fat content and improving aerobic capacity [8,9]. The MedDiet may help reduce outcomes associated with the severity of MASLD, including total cholesterol, liver fibrosis, and waist circumference [10]. Training interventions in MASLD patients, especially aerobic training, have also been associated with reductions in serum levels of the liver enzymes alanine aminotransferase (ALT) and aspartate aminotransferase (AST) [11].

In addition to standard liver enzymes, more specific biomarkers such as cytokeratin-18 (CK-18) have emerged as valuable tools to monitor MASLD progression. CK-18, a hepatocyte intermediate filament protein released into the bloodstream during apoptosis, is widely recognized as a marker for nonalcoholic steatohepatitis (NASH) and MASLD severity [12].

This study adds to the existing literature by evaluating the long-term impact (24 months) of adherence to a Mediterranean lifestyle on hepatic health in patients with MASLD. Unlike prior studies that typically assess short-term effects, this trial offers novel insights into the sustainability and effectiveness of dietary interventions over an extended period. The inclusion of both the Dietary Inflammatory Index (DII) and plasma CK-18 levels provides a more comprehensive picture of inflammatory and apoptotic responses, enhancing the clinical relevance of our findings. Based on this context, this study hypothesizes that improved adherence to the Mediterranean diet, together with increased physical activity, significantly enhanced liver health markers in MASLD patients over a two-year intervention period. Therefore, the objective of this study was to evaluate the effects of a two-year Mediterranean lifestyle intervention, focused on dietary recommendations and physical activity promotion, on liver health parameters in patients with MASLD.

## 2. Methods

### 2.1. Participants and Study Design

The present study involved sixty-two patients diagnosed with MASLD through magnetic resonance imaging, all with Spanish nationality (residing in the Balearic Islands). The participants were selected based on the inclusion criteria outlined in previous research [13]. In short, they were between 40 and 60 years old, had a body mass index (BMI) ranging from 27 to 40 kg/m^2^, and met at least three of the metabolic syndrome (MetS) criteria as defined by the International Diabetes Federation (IDF) consensus [14]. The exclusion criteria were consistent with those described earlier [15]; individuals were excluded if they had a history of cardiovascular disease, congestive heart failure, liver disorders (excluding NAFLD), cancer or malignancy within the past five years, prior bariatric surgery, acute febrile illnesses, urinary tract infections, post-renal hematuria, hemochromatosis, protein overload, untreated depression or anxiety, alcohol or drug abuse, pregnancy, primary endocrine disorders (except hypothyroidism and type 2 diabetes mellitus), ongoing steroid therapy, engagement in intense physical activity, or an inability to provide informed consent. Recruitment took place from 26 October 2018 to 29 November 2019; thus, for some participants the 6- and 12-month time points were affected by the COVID-19 lockdown. During the COVID-19 period, dietary recommendations were made and follow-up was conducted via telephone interviews or video calls and not in person, due to mobility limitations. Anthropometric measurements and biological sample analyses were performed when permitted, using appropriate safety measures and personal protection equipment (EPI), although some delays occurred in some patients.

The study protocol and all procedures were designed in line with the ethical principles of the Declaration of Helsinki and were approved by the Ethics Committee of the Balearic Islands (reference: IB 2251/14 PI). The participants were informed about this study’s objectives and potential risks, and they gave written informed consent to take part. Additional information about the protocol is available on ClinicalTrials.gov under reference NCT0442620 [16].

After being included in the trial, the participants were randomly assigned to one of three groups, as previously described [15]:

1. AASLD Diet (AAD) Group: Participants in this group adhered to dietary guidelines established by the American Association for the Study of Liver Diseases (AASLD), which emphasize caloric restriction to support weight loss. The target was a 3–5% reduction in body weight to alleviate hepatic steatosis, while a 7–10% reduction was recommended to improve histopathological characteristics associated with nonalcoholic steatohepatitis (NASH). The diet followed the nutritional framework outlined by the U.S. Department of Health and Human Services and the U.S. Department of Agriculture. The composition of the diet in this intervention followed the Mediterranean diet pattern, adhering to the following guidelines: consume fresh fruits and vegetables daily, increase fiber intake through whole grains (oats, brown rice, quinoa, whole-grain bread, etc.), use vegetable oils instead of solid fats (almond oil, olive oil, flaxseed oil), limit foods and beverages containing added sugars, eliminate cakes and sweets, choose low-fat dairy products, limit salt intake, prefer skinless white meats, limit the consumption of processed meats (ham, sausages, cold cuts, etc.), and eat fish at least twice a week. The participants ate five meals throughout the day, with macronutrient proportions set at 20–35% fat, 10–35% protein, and 45–65% carbohydrates.
2. Mediterranean Diet with High Meal Frequency (MD-HMF) Group: This group followed a Mediterranean dietary pattern with a structured macronutrient intake: 40–45% carbohydrates, with the majority (50–70%) derived from fiber-rich, low-glycemic sources, 30–35% fat, and 25% protein. The group adhered to the following guidelines: consume seasonal fruits and fresh vegetables daily, eat only whole grains (oats, brown rice, quinoa, whole-grain bread, etc.), use extra-virgin olive oil, limit foods and beverages containing added sugars, eliminate cakes and sweets, choose low-fat dairy products, limit salt intake, prefer skinless white meats, limit the consumption of processed meats (ham, sausages, cold cuts, etc.), consume legumes at least 4 days per week, eat oily fish 2 days per week, and consume nuts every day. Additionally, to optimize energy metabolism, participants consumed seven smaller meals per day, with the highest caloric intake concentrated in the morning hours.
3. Mediterranean Diet with Physical Activity (MD-PA) Group: Participants in this group followed a calorie-controlled Mediterranean diet, with four to five meals per day, including snacks. The dietary composition was the following: 35–40% fat, distributed as 8–10% saturated fats, more than 20% monounsaturated fats, and over 10% polyunsaturated fats, while keeping cholesterol intake below 300 mg/day; approximately 20% protein; 40–45% carbohydrates, primarily sourced from low-glycemic index foods; sodium chloride intake limited to a maximum of 6 g/day (equivalent to 2.4 g of sodium); and dietary fiber intake set at a minimum of 30–35 g/day. Specifically, the guidelines followed in this group were like the previous ones: consume seasonal fruits and fresh vegetables daily, prefer whole grains (oats, brown rice, quinoa, whole-grain bread, etc.), use extra-virgin olive oil, limit foods and beverages containing added sugars, eliminate cakes and sweets, choose low-fat dairy products, limit salt intake, prefer skinless white meats, limit the consumption of processed meats (ham, sausages, cold cuts, etc.), and consume nuts every day.

Regarding physical activity guidelines, the AAD and MD-HMF groups were encouraged to complete a minimum of 10,000 steps daily. The MD-PA group incorporated a structured exercise regimen consisting of a 35 min interval training session, three times per week.

**Figure 1:**
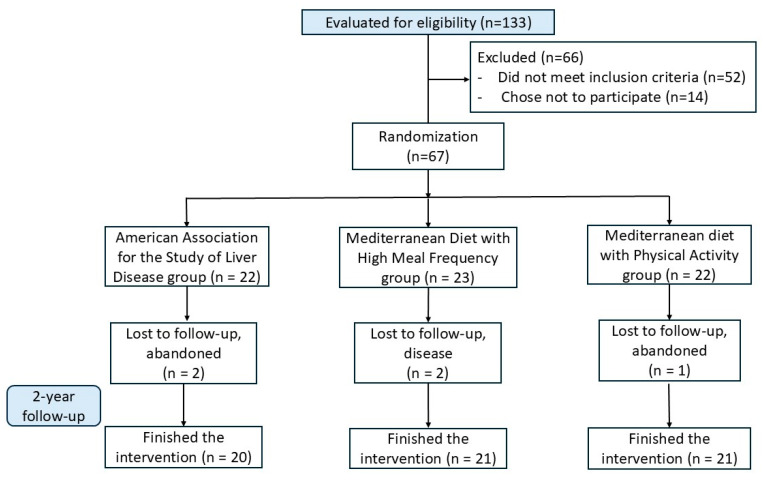
Flow-chart of participants.

A total of 133 patients were assessed for eligibility; 52 did not meet the criteria, and 14 chose not to participate. Ultimately, 67 patients were randomly assigned in equal proportions (1:1:1) to one of the three intervention groups for a two-year period. Unfortunately, five of them could not complete the two-year intervention period; three abandoned it, and two had a disease that prevented them from continuing. The final sample included 62 participants, all those who completed the clinical trial (Figure 1).

The sample size was calculated considering the primary variable, weight loss. The choice of weight loss was made because current AASLD (American Association for the Study of Liver Diseases) recommendations focus solely on achieving weight loss to improve MASLD. According to the results of the RESMENA project [17], a slight difference of 1.0 ± 1.5 kg was expected between the groups with a 95% confidence interval (α = 0.05) and statistical power of 80% (β = 0.8). This calculation estimated that 20 participants per group would be required. Given a dropout rate of 15%, a total of 67 participants were included. Unfortunately, five participants were unable to complete the 24-month follow-up.

In a previously published study, we found that improvements in hepatic health were comparable across all three groups [18]. Based on this, in our previous research, we analyzed short-term adherence-related effects of the Mediterranean diet and lifestyle interventions on hepatic and metabolic health parameters [19,20]. These earlier studies focused on the impact observed within the first six and twelve months of intervention. Given the importance of sustained adherence in achieving meaningful health benefits, this study aimed to evaluate the impact of low and high adherence to MedDiet over a two-year period, studying the 4 time points of the clinical trial.

Throughout the two-year intervention, participants were monitored at each follow-up visit, and any relevant changes in health status were documented. While acute, mild illnesses such as colds or seasonal viral infections were not systematically recorded due to their limited clinical impact, no participant reported serious comorbid conditions or infectious diseases that would compromise the hepatic or metabolic endpoints. Patients with chronic infectious or inflammatory diseases were excluded at baseline.

### 2.2. Adherence to Mediterranean Diet and Diet Inflammatory Index Measurements

Adherence to the MedDiet was evaluated using a validated 17-item MedDiet adherence questionnaire previously utilized [21], which all participants completed at each time point. Each criterion was assigned a score of 1 (compliance) or 0 (non-compliance), resulting in a total score ranging from 0 (no adherence) to 17 (maximum adherence). This 17-item questionnaire has been previously validated in Spanish adult populations [21] and is widely used to assess adherence to the MedDiet in clinical and research settings. As the tool is already validated for the study population and context, no additional pilot testing was conducted in this trial.

The independent variable analyzed was the change in MedDiet adherence after a 24-month follow-up, calculated by subtracting the baseline score from the 24-month score. Based on this, participants were categorized into two groups according to the median value: “low-adherent (<50%)” (*n* = 31) and “highly adherent (≥50%)” (*n* = 31). Classification into these groups was based on changes in the scores from a questionnaire designed to evaluate adherence to the adherence to MedDiet, comparing results at the beginning of the intervention (baseline), after six, twelve, and twenty-four months of intervention.

The DII measures the inflammatory potential of a diet. Dietary intake data were collected using validated FFQs, which were administered by trained personnel at each follow-up point. These data were then used to compute the DII scores as described in the previous literature [22,23]. Briefly, the index evaluates the effects of 45 different foods, nutrients, and bioactive compounds on six inflammatory biomarkers, including C-reactive protein (CRP), four interleukins (IL-1β, IL-4, IL-6, and IL-10), and tumor necrosis factor-alpha (TNF-α). A positive DII score reflects a pro-inflammatory diet, whereas a negative score indicates an anti-inflammatory diet. An individual’s intake for each parameter was adjusted by subtracting the standard mean intake and dividing by its standard deviation (SD). These values were then converted into centered percentile scores and multiplied by their respective inflammatory effect scores. The total DII score was obtained by summing the scores of all food parameters.

### 2.3. Anthropometrics Measurements and Chester Step Test

Body weight was measured using a Segmental Body Composition Analyzer (Tanita BC-418, Tanita, Tokyo, Japan) while participants wore light clothing and no shoes, and 0.6 kg was subtracted to account for clothing weight. Height was measured to the nearest millimeter using an anthropometer (Seca 214, SECA Deutschland, Hamburg, Germany). The body mass index (BMI) was then calculated as body weight in kilograms divided by height in square meters (kg/m^2^). To reduce measurement variability between observers, trained personnel carried out the anthropometric assessments. The Chester step test was used to measure the maximal oxygen uptake (VO_2_max) [24].

### 2.4. Blood Collection and Biochemical Parameters

Blood samples were obtained from all participants after 12 h overnight fast. The samples were collected in tubes containing ethylenediaminetetraacetic acid (EDTA) and then centrifuged at 1700× *g* for 15 min at 4 °C. Biochemical parameters, including glucose, triglycerides (TG), AST, ALT, and gamma-glutamyl transferase (GGT), were measured following standard clinical protocols [15].

### 2.5. Hepatic Parameters

Intrahepatic fat content (IFC) measurements were conducted using a 1.5-T MRI scanner (Signa Explorer 1.5T, General Electric Healthcare, Chicago, IL, USA) equipped with a 12-channel phased-array coil [25]. The fatty liver index (FLI) was calculated using the following formula [26]:

$$\mathrm{Fatty}\mathrm{Liver}\mathrm{Index}\left(FLI\right)=\frac{e^{y}}{\left(1+e^{y}\right)}\times 100$$

where

$$y=0.953\times \ln\left(TG\right)+0.139\times BMI+0.718\times \ln\left(GGT\right)+0.053\times WC-15.745$$

The abbreviations and units used are TG for triglycerides (mg/dL), BMI for the body mass index (kg/m^2^), GGT for γ-glutamyl transferase (U/L), and WC for waist circumference (cm).

Cytokeratin-18 (CK-18) levels in plasma were measured using the M30 Apoptosense^®^ ELISA kit, following the manufacturer’s instructions (PEVIVA^®^, DiaPharma Group, Inc., P.O. Box 32160, Department 101, Louisville, KY 40232-2160, USA).

### 2.6. Statistics

Statistical analysis was conducted using the Statistical Package for the Social Sciences (SPSS v.29 for Windows, IBM Software Group, Chicago, IL, USA).

The results were presented as mean (standard deviation; SD), with statistical significance set at *p* < 0.05 for all analyses. The distribution of continuous data was assessed using histograms and normal probability plots to determine its conformity to a normal distribution. Baseline comparisons between the low-adherent and highly adherent groups were performed using an independent sample t-test for continuous variables (e.g., age) and a chi-square test for categorical variables (e.g., sex). Additionally, a multivariate general linear model was used to assess statistical significance, adjusting for age, sex, and intervention (diet and physical activity). Bonferroni’s post hoc test was employed to identify differences between groups and between time points within the same group. Additionally, Pearson correlation analyses were conducted to assess the relationship between the change in MedDiet adherence and changes in hepatic health parameters. Change scores were calculated by subtracting baseline values from 24-month follow-up values.

The as-treated principle was applied in the statistical analysis. Rather than analyzing participants based on their originally assigned intervention groups, they were categorized according to their adherence to the MedDiet throughout the study period, as explained in the Adherence to MedDiet section. This approach provided a more accurate assessment of the real impact of dietary adherence on hepatic health parameters in MASLD patients. By focusing on adherence to MedDiet rather than initial randomization, these findings better reflect the effectiveness of long-term lifestyle interventions on liver health outcomes.

## 3. Results

### 3.1. Characteristics of Participants

At baseline, the low-adherent group (*n* = 31) had a mean age of 54.3 ± 6.5 years and included 15 women (48%). The highly adherent group (*n* = 31) had a mean age of 50.6 ± 6.6 years and included 16 women (52%). No statistically significant differences were observed between groups in terms of age or sex distribution. Moreover, at baseline, no significant differences were observed between groups in anthropometric and biochemical parameters. However, significant differences were found in adherence to the MedDiet and the DII between groups at baseline comparison, which was expected since these variables defined the grouping criteria of this study.

Table 1 shows the characteristics of participants stratified by adherence to the MedDiet. A reduction in weight was observed at the different sampling points compared to baseline in both groups, except in the low-adherent group, where after 24 months there was an increase in weight, recovering to initial weight values. Adherence to the MedDiet was significantly higher at the 6-, 12-, and 24-month time points than at baseline in both groups. BMI was significantly lower in the highly adherent group at each time point compared to at baseline, while in the low-adherent group, after an initial drop at 6 months, BMI increased, recovering to initial values.

The DII significantly improved in participants with better adherence to the MedDiet, with values at 6, 12, and 24 months in higher-adhering participants being significantly different from those at baseline. In the low-adherent group, the DII also significantly decreased at all time points compared to those at baseline. At the 24-month time point, there was a significant difference in the DII between the participants with higher adherence and lower adherence to the MedDiet; participants who did not adhere to the MedDiet reflected a pro-inflammatory diet, whereas those who improved their adherence presented an anti-inflammatory diet.

Although no significant differences were observed in glucose, triglycerides, and VO₂max (measured via the Chester step test), effect sizes were calculated for these three parameters across all time points. It was found that the partial eta squared (η^2^ partial) for most parameters was below 0.01. However, adherence to the MedDiet had a small effect on triglycerides at 6 months (η^2^ = 0.015; *p* = 0.096). The effect on the Chester step test was also small, with the highest impact observed at 24 months (η^2^ = 0.034; *p* = 0.026). In the case of glucose levels, adherence to the Mediterranean diet did not have a meaningful impact in this study.

Regarding potential confounders, the number of participants using antihypertensives (40% of low-adherent and 40% of highly adherent participants), hypoglycemic medications (10% of low-adherent and 5% of highly adherent participants), and hypolipidemic medications (40% of low-adherent and 20% of highly adherent participants) showed no significant difference between the two groups, based on the statistical analysis of the data. This indicates that medication is likely to have little impact on the adherence to MedDiet through lifestyle intervention.

### 3.2. Liver Enzyme Levels

Table 2 shows the liver enzyme levels of participants with MASLD at four follow-up time points stratified by adherence to MedDiet. AST levels significantly decreased in the group in which one improved their adherence to the MedDiet, with a notable reduction observed after six months of intervention compared to at baseline. Additionally, ALT and GGT levels showed significant differences between groups and time points.

### 3.3. Hepatic Health Parameters

Table 3 shows the IFC levels, FLI, and plasma CK-18 levels in participants with MASLD according to their improvement in adherence to the MedDiet. The IFC levels improved significantly in the group with a higher adherence to the MedDiet at the time points of 6, 12, and 24 months compared to at baseline, although a loss of significance was observed at 12 months, derived from the period of confinement due to COVID-19. In contrast, the IFC levels in the group with low adherence to the MedDiet showed a non-significant decrease at 6 months, recovering to initial values at 12 and 24 months. In fact, the values of IFC at 24 months were significantly higher than those at 6 months. FLI values significantly decreased in the group with better adherence to the MedDiet at each time point, compared to at baseline, whereas they remained unchanged in the group with lower adherence to the MedDiet. The FLI presented significant differences between groups, shown at the 24-month time point. Plasma CK-18 levels were significantly reduced in the highly adherent group after 6, 12, and 24 months compared to at baseline. In contrast, in the low-adherent group, after an initial non-significant downward trend, CK-18 levels increased, particularly at the 12-month time point, which coincided with the onset of the COVID-19 pandemic. Additionally, CK-18 levels showed significant differences between groups at baseline, 12 months, and 24 months.

To further explore the relationship between dietary behavior and liver health, a correlation analysis was performed between the change in adherence to the MedDiet and the change in hepatic parameters over the 24-month intervention period. The change in MedDiet adherence was calculated as the difference in adherence scores from baseline to 24 months.

The results showed statistically significant negative correlations between improved MedDiet adherence and reductions in intrahepatic fat content (IFC) (r = −0.42; *p* < 0.01), the fatty liver index (FLI) (r = −0.39; *p* < 0.05), and plasma cytokeratin-18 (CK-18) levels (r = −0.44; *p* < 0.01) (Table 4). These findings suggest that a greater increase in adherence to the MedDiet is associated with a more pronounced improvement in hepatic health markers in patients with MASLD.

### 3.4. Impact of COVID-19 on Adherence and Results

The COVID-19 period likely impacted adherence to the MedDiet due to limited access to fresh foods, disrupted routines, and reduced physical activity, leading to increased consumption of processed foods. Lockdowns and social distancing also affected eating habits by reducing communal meals. However, some participants improved their adherence due to increased health awareness and telephone follow-ups by dietitians. Notably, after the COVID-19 period ended, participants who maintained better adherence to the MedDiet showed improved parameters at the 24-month session.

## 4. Discussion

The current study provides significant evidence on the benefits of adherence to the MedDiet in improving liver parameters in patients with MASLD. After a two-year follow-up, patients who improved their adherence to the MedDiet experienced reductions in systemic inflammation, IFC, the FLI, and plasma levels of CK-18, a key biomarker in the progression of fatty liver disease.

These findings are consistent with results from recent 12-month clinical trials that have evaluated Mediterranean diet interventions in MASLD populations. It was reported that a Mediterranean diet-oriented intervention, even without caloric restriction, restored mitochondrial function in peripheral blood mononuclear cells, indicating systemic metabolic improvements [27]. Significant reductions in liver steatosis and anthropometric parameters were observed following a Mediterranean-oriented dietary plan in Italian patients [28]. Although both studies covered a shorter intervention period than ours, their results reinforce the efficacy of the Mediterranean dietary pattern in improving hepatic and metabolic health in MASLD.

A significant reduction in body weight and BMI was observed in the group with higher adherence to the MedDiet. This aligns with previous research, which has shown that the MedDiet, being rich in low-glycemic-index foods and healthy fats, facilitates weight loss and improves insulin sensitivity, contributing to a reduction in hepatic lipid load [29,30]. The MedDiet’s richness in fruits, vegetables, whole grains, and healthy fats has been shown to lower liver fat, inflammation, and oxidative stress—key factors in MASLD progression [31,32]. Additionally, it supports cardiovascular health, metabolic function, and sustainable lifestyle changes, potentially reducing the risk of obesity-related conditions such as type 2 diabetes and cardiovascular disease [33,34].

A key finding of this study is the significant reduction in DII in patients with higher adherence to MedDiet over two years of follow-up. This reduction was observed progressively across the four follow-up sessions. In the current cohort, improved adherence to an anti-inflammatory diet, as reflected by lower DII scores, was associated with significant reductions in body weight and visceral fat, alongside improvements in cardiometabolic and inflammatory profiles. These findings are consistent with previous studies that reported similar associations between anti-inflammatory dietary patterns and improved anthropometric and metabolic outcomes in individuals with obesity [9,33]. Moreover, participants with lower DII values exhibited a significantly reduced risk of fatty liver disease and liver fibrosis compared to those with higher DII [35]. Additionally, the decrease in DII in participants with greater adherence to the MedDiet suggests a beneficial effect on chronic inflammation regulation. This is particularly relevant, as inflammation plays a crucial role in the progression of MASLD to more severe stages, such as NASH. The reduction in inflammatory markers may be driven by the high levels of polyphenols and monounsaturated fatty acids present in the MedDiet [36,37].

Reductions in hepatic enzymes like AST, ALT, and GGT in patients following the MedDiet are in accordance with previous studies [38,39]. Since these enzymes are key indicators of liver dysfunction, the findings suggest that stronger adherence to the MedDiet can significantly reduce liver damage [40]. This reduction also suggests a direct correlation between dietary patterns and liver enzyme levels. AST levels showed a steady decline over the follow-up period in the high-adherence group. ALT and GGT reductions indicate improved hepatocellular function and reduced liver inflammation. These findings support the existing literature on the MedDiet’s hepatoprotective effects through oxidative stress reduction and metabolic improvement [41,42].

While dietary modifications significantly influenced hepatic health, the role of physical activity must not be overlooked. The increase in maximal oxygen uptake (VO_2_max) in the high-adherence group suggests an improvement in cardiorespiratory fitness, which has been previously associated with enhanced hepatic metabolism and reduced liver fat [43]. Engaging in structured physical activity, particularly aerobic exercise, has been shown to reduce serum levels of ALT and AST, both of which were observed in this study. These findings reinforce that integrating physical activity with dietary interventions yields optimal outcomes in MASLD management [44].

Regarding hepatic parameters in this study, a significant reduction in IFC was observed after 24 months of intervention compared to at baseline. However, the greatest reduction occurred between baseline and after six months of intervention. A similar pattern was observed in the FLI among responder participants who adhered to the MedDiet. The FLI was calculated as an indicator to assess the severity of fatty liver and the risk of cardiovascular disease [45,46]. These reductions suggest that patients improved their MASLD after 24 months of following MedDiet recommendations and engaging in physical activity. In this context, an association between MedDiet and improved liver status has been observed in patients with overweight, obesity, and MASLD [47].

CK-18 is a valuable plasma biomarker for identifying and monitoring MASLD [45], which was evaluated in this study. Additionally, CK-18 fragment levels correlate with the magnitude of hepatocyte apoptosis, providing insight into liver cell death and inflammation [48]. A notable finding is the significant reduction in CK-18 levels observed in highly adherent participants; this reduction was more pronounced after 6 and 24 months of intervention, with some impact likely due to the COVID-19 pandemic occurring between the 6- and 12-month sessions. Previous evidence supports these results, showing that better adherence to the MedDiet positively impacted CK-18 levels, which were reduced in the group with higher adherence to MedDiet after intervention [19,20]. Lower CK-18 levels suggest improved liver health, such as reduced hepatocyte apoptosis and necrosis, reflecting a potential improvement in liver function [49]. Additionally, lower CK-18 levels may indicate reduced inflammation and fibrosis risk, as CK-18 is associated with liver injury and inflammation. Therefore, its reduction may imply a lower risk of progression to fibrosis or cirrhosis [50].

From a clinical standpoint, the findings highlight the potential for lifestyle modifications, particularly MedDiet adherence and increased physical activity, as non-pharmacological interventions for MASLD. Given that MASLD currently lacks approved pharmacological treatment, dietary and lifestyle interventions remain the cornerstone of management [51]. Clinicians should emphasize a multidisciplinary approach, integrating nutritional counseling, structured exercise programs, and behavioral support to optimize patient outcomes. In particular, the integration of the MedDiet with physical activity offers a promising strategy for managing MASLD and mitigating its progression.

The observed benefits of MedDiet adherence can be partly attributed to its high content of polyphenols and unsaturated fats, which exert hepatoprotective effects through multiple mechanisms. Polyphenols, abundant in olive oil, fruits, and vegetables, have been shown to modulate oxidative stress, reduce inflammatory cytokine levels, and enhance mitochondrial function [52]. Additionally, unsaturated fats, particularly monounsaturated fatty acids (MUFA) and omega-3 polyunsaturated fatty acids (PUFA), contribute to the regulation of hepatic lipid metabolism by improving insulin sensitivity and reducing lipotoxicity [53]. These effects may collectively attenuate hepatocellular damage, reduce fibrosis progression, and improve overall liver function.

Although the current statistical models were adjusted for sex to minimize the potential confounding effects of gender on clinical outcomes, the influence of sex differences in MASLD progression and response to intervention remains an important consideration. The literature suggests that sex hormones and metabolic distinctions between men and women significantly impact the prevalence and severity of steatotic liver disease. For instance, MASLD is generally more prevalent in men than in premenopausal women, likely due to the hepatoprotective effects of estrogen [46]. This difference tends to diminish post-menopause, as estrogen levels decline. Additionally, men and women may exhibit distinct metabolic responses to lifestyle interventions, with men often showing greater reductions in visceral fat, whereas women may demonstrate more favorable changes in lipid profiles and systemic inflammation [42,47]. These sex-related physiological variations may influence the efficacy of dietary and physical activity strategies in managing MASLD. Future studies should incorporate sex-specific analyses to better understand differential responses and to support the development of tailored intervention strategies.

The novelty of the current study lies in its two-year duration and comprehensive evaluation of hepatic health in MASLD patients following a Mediterranean lifestyle intervention. Unlike most previous studies limited to short-term effects, the current findings provide novel insights into the long-term sustainability and clinical benefits of MedDiet adherence. Moreover, the use of both the DII and plasma CK-18 levels enhances current understanding of the inflammatory and apoptotic responses in relation to lifestyle change, supporting the role of diet as a central element in MASLD management.

### Strengths and Limitations

The primary strength of this study is the significant improvement of hepatic health parameters in patients with MASLD, especially in those who adhered higher to adherence to MedDiet. However, a disruption in improvements at the 12-month follow-up due to the COVID-19 pandemic was noted. Lockdown restrictions likely affected participants’ dietary adherence and physical activity levels, underscoring the importance of sustained lifestyle interventions in managing chronic metabolic conditions. One limitation of this study is the relatively small sample size. However, despite this limitation, the sample was large enough to detect significant differences in hepatic health parameters between individuals with high and low adherence to the MedDiet. Additionally, since the participants were aged 40 to 60, the findings may not be directly applicable to younger populations, and caution should be exercised when generalizing the results beyond this age group. Future studies should consider these age-related differences to broaden the applicability of the findings to a more diverse demographic. A limitation of this study is the lack of adjustment for potential confounders such as medication use and comorbidities, which may have influenced the results. Another limitation of this study is the use of a median split to categorize participants into high- and low-adherence groups. While this method is widely applied in nutritional epidemiology, it may result in participants with very similar adherence scores being placed into separate categories, potentially reducing the ability to detect gradual, dose–response relationships in dietary adherence and hepatic outcomes. Despite these limitations, the findings provide compelling evidence for the role of long-term adherence to the Mediterranean diet, alongside physical activity, as an effective non-pharmacological strategy for improving hepatic and metabolic health in MASLD patients.

## 5. Conclusions

Adherence to the MedDiet can be enhanced, leading to significant improvements in hepatic health parameters and overall lifestyle in patients with MASLD. Beyond liver-specific benefits, this dietary pattern supports metabolic and cardiovascular health, reinforcing its role in comprehensive disease management. Future research should explore tailored strategies to improve long-term adherence, including structured dietary counseling and behavior-focused interventions. Additionally, integrating the MedDiet into MASLD treatment protocols should be further investigated, assessing its feasibility in clinical settings and the potential need for personalized dietary adaptations based on patient characteristics.

## Figures and Tables

**Table 1:** Characteristics of participants at four follow-up time points stratified by adherence to Mediterranean diet.

		Low-Adherent (<50%)				Highly Adherent (≥50%)				*p*-Value
	Reference Values	Baseline (*n* = 31)	6 Months (*n* = 31)	12 Months (*n* = 31)	24 Months (*n* = 31)	Baseline (*n* = 31)	6 Months (*n* = 31)	12 Months (*n* = 31)	24 Months (*n* = 31)	
Sex (women, *n*[%])		15 (48%)				16 (52%)				0.793
Age (years)		54.3 (6.5)				50.6 (6.6)				0.051
Weight (kg)		96.1 (14.4)	93.1 (15.0)	93.4 (15.1)	93.8 (18.6)	92.4 (13.7)	87.89 (12.3)	88.6 (12.7)	89.5 (13.0)	0.443
BMI (kg/m^2^)		33.7 (3.31)	32.6 (3.31)	32.7 (3.49)	33.5 (3.5)	33.4 (4.44)	31.7 (3.89)	31.9 (4.09)	32.1 (3.79)	<0.001
Glucose (mg/dL)	70–110	110.1 (22.0)	107.9 (36.0)	107.5 (26.6)	108.4 (21.6)	108.8 (19.1)	103.1 (18.2)	102.4 (18.4)	105.9 (23.8)	0.278
Triglycerides (mg/dL)	<149	178 (63)	186 (98)	173 (68)	166 (45)	201 (79)	172 (97)	179 (78)	170 (47)	0.884
Bilirubin (mg/dL)	0.2–1.2	0.71 (0.39)	0.77 (0.53)	0.76 (0.34)	0.70 (0.33)	0.74 (0.38)	0.77 (0.32)	0.74 (0.26)	0.74 (0.26)	0.944
ADM		9.5 (2.4)	11.8 (2.5) ^a^	11.0 (2.6) ^b^	10.9 (2.6) ^c^	6.9 (2.4) *	12.2 (2.9) *	12.5 (2.2) ^b,^*	12.6 (2.0) ^c,^*	<0.001
DII		0.4 (2.2)	0.2 (2.4) ^a^	0.2 (2.2) ^a^	0.3 (2.2) ^a^	0.5 (2.4) *	−0.13 (2.0) ^a^	−0.3 (2.2) ^a^	−0.52 (1.9) ^a,^*	<0.001
Chester step test (VO_2_max)		32.8 (7.9)	34.0 (6.1)	35.7 (7.8)	34.6 (6.1)	34.4 (8.8)	34.6 (9.4)	37.1 (8.3)	37.3 (10.8)	0.449

Abbreviations: ADM: adherence to Mediterranean diet; DII: Dietary Inflammatory Index. Data are shown as the mean (standard deviation) for continuous variables and number (and percentage) for categorical variables. A chi-square test was used to compare sex distribution between groups, and an independent sample *t*-test was used to compare baseline age. Other variables were analyzed using a multivariate general linear model after adjustments by age, sex, and intervention (diet and physical activity). Bonferroni: ^a^ differences with respect to baseline, ^b^ differences with respect to 6 months, ^c^ differences with respect to 12 months. * Differences in means between groups at the same time point. Data points are considered significant when *p* < 0.05.

**Table 2:** Liver enzyme levels of participants at four follow-up time points, stratified by adherence to Mediterranean diet.

		Low-Adherent (<50%)				Highly Adherent (≥50%)				*p*-Value
	Reference Values	Baseline (*n* = 31)	6 Months (*n* = 31)	12 Months (*n* = 31)	24 Months (*n* = 31)	Baseline (*n* = 31)	6 Months (*n* = 31)	12 Months (*n* = 31)	24 Months (*n* = 31)	
		Mean (SD)	Mean (SD)	Mean (SD)	Mean (SD)	Mean (SD)	Mean (SD)	Mean (SD)	Mean (SD)	
AST (U/L)	5–34	21.8 (6.0)	20.8 (6.7)	20.0 (5.7)	24.0 (8.2) ^b^	29.7 (17.7) *	24.2 (8.1) ^a,^*	24.9 (9.1) *	25.8 (10.4)	0.036
ALT (U/L)	0–55	33.9 (19.0)	26.5 (14.8)	25.5 (11.3)	31.3 (17.2)	43.5 (28.9)	30.4 (11.8)	33.2 (18.6)	35.8 (18.3)	0.104
GGT (U/L)	12–64	42.5 (23.5)	35.9 (22.0)	37.8 (21.8)	43.7 (33.9)	50.2 (49.6)	34.3 (19.1)	35.3 (15.2)	35.6 (15.4)	0.055

Abbreviations: AST: aspartate aminotransferase; ALT: alanine aminotransferase; GGT: gamma-glutamyl aminotransferase. Multivariate general linear model after adjustments by age, sex, and intervention (diet and physical activity). Bonferroni: ^a^ differences with respect to baseline, ^b^ differences with respect to 6 months. * Differences in means between groups. Data points are considered significant when *p* < 0.05.

**Table 3:** Hepatic health parameters of participants at four follow-up time points, stratified by adherence to Mediterranean diet.

	Low-Adherent (<50%)				Highly Adherent (≥50%)				*p*-Value
	Baseline (*n* = 31)	6 Months (*n* = 31)	12 Months (*n* = 31)	24 Months (*n* = 31)	Baseline (*n* = 31)	6 Months (*n* = 31)	12 Months (*n* = 31)	24 Months (*n* = 31)	
	Mean (SD)	Mean (SD)	Mean (SD)	Mean (SD)	Mean (SD)	Mean (SD)	Mean (SD)	Mean (SD)	
IFC (%)	13.7 (8.08)	10.2 (7.26)	12.1 (8.05)	13.5 (8.54) ^b^	18.4 (13.4)	12.0 (7.09) ^a^	12.7 (7.62)	12.5 (5.8) ^c^	0.038
FLI	88.3 (11.3)	85 (11.5)	84.5 (10.4)	87.9 (9.1)	89.8 (10.1)	79.1 (18.2) ^a^	80.2 (16.4) ^a^	82.3 (13.1) ^a,^*	0.002
CK-18 (U/L)	52.6 (28.1)	44.6 (30)	65.6 (35.7) ^b^	52.6 (45.2)	75.7 (47.0) *	45.1 (26.7) ^a^	45.8 (23.9) ^a,^*	31.6 (16.7) ^a,^*	<0.001

Abbreviations: IFC: intrahepatic fat content; FLI: fatty liver index; CK-18: cytokeratin 18. Multivariate general linear model after adjustments by age, sex, and intervention (diet and physical activity). Bonferroni: ^a^ differences with respect to baseline, ^b^ differences with respect to 6 months. * Differences in means between groups. Data points are considered significant when *p* < 0.05.

**Table 4:** Pearson correlation coefficients between changes in MedDiet adherence scores and changes in hepatic parameters from baseline to 24 months.

Hepatic Health Parameter	*r*	*p*-Value
IFC	−0.422	<0.001
FLI	−0.393	0.005
CK-18	−0.444	<0.001

Abbreviations: IFC: intrahepatic fat content; FLI: fatty liver index; CK-18: cytokeratin 18. Correlations are based on change scores calculated as 24-month value minus baseline value. Negative correlations indicate that greater improvements in MedDiet adherence were associated with reductions in hepatic risk markers.

## Data Availability

Access to the trial data is restricted due to signed consent agreements, which permit data sharing only for studies aligned with the project’s objectives. Researchers interested in accessing the data used in this study can submit a request to pep.tur@uib.es.
